# Forming Homogeneous Three-Dimensional Structures from Discrete Silica Microspheres Using Sub/Supercritical Water

**DOI:** 10.1021/acsami.4c07251

**Published:** 2024-08-01

**Authors:** Pavel Karásek, Josef Planeta, Michal Roth

**Affiliations:** Institute of Analytical Chemistry of the Czech Academy of Sciences, Veveří 97, 60200 Brno, Czech Republic

**Keywords:** silica microspheres, supercritical water, homogeneous three-dimensional structure, close-packed bed, liquid chromatography, monolithic column

## Abstract

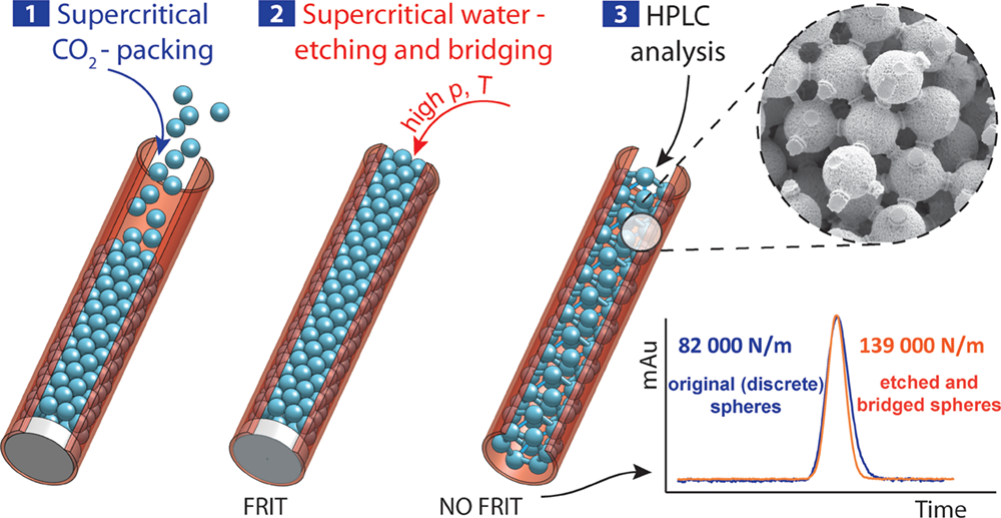

A novel technique for producing highly uniform structures from silica microspheres has been developed and tested. It is based on exploiting the temperature- and pressure-dependent solvent properties of sub/supercritical water toward silicon dioxide. The initial concept aimed to create a “hybrid” capillary chromatographic column on the border between a packed and a monolithic column that would combine the benefits of both. The resultant method that integrates dissolution and coalescence in a continuous process enabled the production of a range of permeable columns with high efficiency and varying sizes. Their internal structures were examined using scanning electron microscopy and characterized using microHPLC chromatography. The structures produced using this method may have diverse applications beyond the scope of analytical chemistry. They prove useful in scenarios where high pressure is necessary because of the high hydraulic resistance of small particles and/or the passing medium with a high flow rate. A simple test of a bridged-microsphere monolithic column and a discrete microsphere-packed column, both after chemical modification to the C18 stationary phase, indicated superior performance of the new type of monolithic columns.

## 1 Introduction

3D porous monolithic structures play an important role in many fields of science and technology.^1^ A relatively small but important application is in analytical separations by liquid chromatography.^2,3^ During the intense development of column chromatography over the past decades, monolithic columns have become a worthwhile alternative to packed columns, with each of the two column classes featuring the pros and cons of its own.^2,4,5^ Traditionally, the build-up material for monolithic columns can be either various organic monomers^6^ or silica^7,8^ although hybrid organic–silica monolithic columns have been gaining ground recently.^9^ Compared to the particle-packed columns, the monolithic columns are usually more permeable, and they also offer some technical advantages over the packed columns as the monoliths do not require the column end frits. In the capillary column format, monolithic silica columns may provide efficiency exceeding that of monolithic organic polymer columns. On the contrary, the monolithic polymer columns are usually easier to prepare as compared to the monolithic silica columns. Overall, given the adverse geometry of the prospective capillary column characterized by a very high length-to-diameter ratio, it is clear that the preparation of a uniform monolithic structure along the entire length of the capillary is not an easy task.

Capillary monolithic columns for liquid chromatography, whether silica-based, organic, or hybrid organic–silica, have usually been prepared from an initially homogeneous mixture, often by the sol–gel method^10^ or by radical polymerization. The mixture is filled into the tube to become the future column, and subsequently converted into the monolithic structure inside the tube.

In this contribution, we present an alternative route to siliceous monoliths that starts from discrete spherical particles of silica. A fused silica capillary is packed with silica microspheres, the void space of the packed capillary is filled with water, and the microspheres are subsequently fused together by temporary action of in situ generated near- or supercritical water (SCW). As SCW is known to solubilize both quartz^11,12^ and amorphous silica,^13^ it has the potential to redistribute the material of silica microspheres in such a way as to reduce the microsphere diameter and form connecting bridges between the nearest neighbor microspheres. The connecting bridges are also formed between the near-wall microspheres and the column wall; this feature is highly important for the efficiency of the resultant column. Further, the SCW-induced improvements in important chromatographic characteristics such as column efficiency (plate height), column permeability, external porosity, and separation impedance are evaluated and discussed.

What prompted us to pursue this particular application of SCW? In the past decade, we have used SCW to treat the inner surfaces of fused silica capillaries^14^ prior to their application in electromigration separations.^15−18^ The ability of SCW to convert close packed silica microspheres into a 3D monolithic structure has been revealed during our attempt to employ SCW to modify the surface structure of silica microspheres packed into a fused silica capillary.

Finally, it should be emphasized that any chemical modification to impart a desired selectivity can only be applied after the SCW treatment of the column has been completed and the bridges already formed. This is because the action of SCW would certainly strip off any surface-bonded chemistry if the treatment with SCW were applied only after the chemical modification.

## 2 Materials and Methods

### 2.1 Chemicals and Materials

Commercial silica microspheres sized 5.04 μm with nonporous surface (Polysciences Europe GmbH, Germany) were used as received. Both, undeactivated fused silica capillary for the column (100 μm i.d., 365 μm o.d.) and 10 μm i.d., 365 μm o.d. for the restrictor were purchased from Agilent Technologies, Germany. CO_2_ for column packing in purity 4.8 was obtained from SIAD, Czech Republic. For HPLC analysis, the miliQ quality water was used, purity of acetonitrile (Fischer Scientific, Czech Republic) was LC/MS grade (Optima). Test compounds uracil, benzene, toluene, ethylbenzene, propylbenzene, butylbenzene, pentylbenzene, and hexylbenzene were purchased from Sigma-Aldrich (Austria).

Chlorodimethyl-octadecylsilane (95%) was obtained from Sigma-Aldrich (Austria). Diethylamine (99.5%) was purchased from Fluka (Belgium).

Preparation of water for the SCW experiment consists of double distillation followed by reverse osmosis (Ultra Clear UV, SG Wasseraufbereitung and Regenerierstation, Barsbüttel, Germany). Finally, the water was stripped with a gentle stream of helium (purity 4.8, Siad Czech) for removing the rest of dissolved gases.

### 2.2 Micropacked Column Preparation

First, a porous ceramic frit was prepared at one end of the fused silica capillary (i.d. 100 μm). We used a simplified version of the procedure described by Cortes.^19^ Briefly, six volumes of water glass solution (34–38% Na_2_SiO_3_, Kittfort Prague, Czech Republic) was mixed with three volumes of water and one volume of formamide (purum, Lachema n.p. Brno, Czechoslovakia). The coagulated mix was stirred until a homogeneous solution was formed (10–15 min). End of the fused silica capillary was immersed in the liquid and the solution was sucked up by capillary elevation (20–30 mm). This capillary end was plugged by silicone septum and put in the oven at 80 °C for 10 min. Then, the silicone plug was cut off and the frit was dried in an oven at 80 °C overnight. Next, end of the capillary with the frit was connected with a flow restrictor (5 μm i.d./10 cm length). The other end of the capillary was connected with a thick-walled stainless-steel reservoir (5 mm i.d., 10 cm length), containing approximately 20–30 mg of the silica microspheres. The reservoir was connected through a needle valve with a high-pressure syringe pump HPP 5001 (Laboratory Instruments, Prague, Czech Republic), filled with liquid CO_2_.

At the beginning of the filling procedure, the reservoir was installed vertically in the holder in a restrictor-up position and the valve was slowly opened to prevent swirling of the silica microspheres in the reservoir and uneven packing. Then, the reservoir was turned 180° so that the restrictor was positioned downward and the whole fused silica capillary was immersed in water (50 °C) in an ultrasonic bath (Bandelin Sonorex, RK 52 H, Germany). Time to complete the filling was about 20–30 min at a pressure of 20 MPa. Then, the column was pulled out of the bath, the valve was closed, and the column was allowed to depressurize spontaneously at ambient temperature for 12 h. Details of this filling method and the results obtained for the preparation of capillary LC columns were described before.^20^

ODS modification of silica particles was performed according to Tanaka.^21^ It uses ODS-DEA to create a monolayer of the C18 stationary phase on the silica support. In brief, 2.0 g of chlorodimethyl-octadecylsilane was dissolved in 7.5 mL of dry toluene, and 1.5 mL of diethylamine was slowly added. The mixture was stirred and after 2 h of reaction, liquid solution was separated by centrifuge. This, approximately 20% solution of ODS-DEA was used for column particle modification at 80 °C for 15 h at a flow rate of 6 μL/h. After modification, the column was thoroughly washed with dry toluene and methanol.

### 2.3 Equipment for Simultaneous Generation and Application of SCW

Three experimental approaches were employed to create a uniform structure through the etching of silica spheres and the connection of bridges, which included one static and two dynamic modes with regard to SCW flow. The static mode, wherein the packed capillary is pressurized with water and subsequently heated to supercritical temperature, was deemed ineffective due to the relatively low solubility of SiO_2_ in SCW. It led to only a slight disruption of the outer surface structure of the spheres. The second method involved a continuous supply of water to the heated capillary. It produced partial desired results at the column inlet. However, the impact lessened with an increasing distance from the inlet. Despite resulting in a 3D structure, its homogeneity remained very low.

The third mode of dynamics, involving both water flow and capillary movement, relied on the creation of SCW within a very short heated metallic block through which the capillary was continuously moved. The capillary displacement was counter-currently oriented with respect to the direction of the water flow, and all the processes, including SCW formation, SiO_2_ dissolution, and consequent SiO_2_ recrystallization, occurred simultaneously and exclusively within this short section.

The apparatus employed to treat the packed fused silica capillaries with SCW was a modified version of the in-lab-assembled setup used before to treat the inner surface of the capillaries prior to their use in electromigration separations.^14−18^

The density of water as a function of temperature and pressure was calculated using a software package^22^ including high-precision thermodynamic formulation for water.^23^

### 2.4 Capillary Handling: Workflow

The FS capillary with an internal diameter of 100 μm packed with 5 μm microspheres (packing procedure is described in detail in Section 2.2) has a total length of 60 cm, of which 30 cm is made up of the microsphere packing. In the first step, the end of the capillary with the packing (closed with a frit) is fixed into a Valco Izera 1.5 high-pressure coupling (SI, Phase2,14), which is fitted with a restrictor (SI, Phase2,7) at the other end, consisting of a FS capillary with a diameter of 5–10 μm. The length and diameter of the restrictor are chosen to achieve the desired SCW flow rate (in text) at a given pressure (in text) in the system. The capillary is then passed through a heater with a working length of 10 mm and a diameter of 0.4 mm (SI, Phase2,13), followed by pulling through the guide tubes between the rubberized wheels of the linear moving device (SI, Phase2,12) and finally reattachment to the Valco Izera 1.5 high-pressure coupling installed directly on the body of the Sensirion SLG0064 precision microflowmeter (SI, Phase2,11). In this initial setup, the heater is localized outside the microparticle bed itself, in the empty part of the capillary. The experiment starts by switching on the high-pressure pump and reaching the set pressure (SI, Phase2,3). The system is allowed to equilibrate for about 5–10 min, and the size of the flow is monitored while the capillary is slowly moved counter-currently at a rate of 1–3 mm/min. In the case of a low flow rate, this is increased by shortening the restrictor. After the flow rate has stabilized, heating is switched on (still in the empty part of the capillary) and after the desired temperature is reached, the capillary is moved at a fast-moving rate about 1 cm before the beginning of the packed part. The capillary linear moving rate at which all columns in this work were subsequently etched was set at 0.79 mm/min, and the final length of the prepared monolith ranged from 20 to 25 cm. To terminate the experiment, the heating was stopped, the system was depressurized by releasing the frit end from the high-pressure coupling, and the capillary was quickly removed from the device so as not to plug it by precipitation of dissolved SiO_2_. The high-pressure pump was left running for about 10 min yet and the capillary was flushed with clean water. Subsequently, the empty part of the capillary was cut off, as well as the rest of the unetched part of the packing, and the monolith thus prepared was then directly subjected to chromatographic analysis or chemically modified by ODS. Part of the monoliths were cut into approximately 3 cm pieces and their geometric homogeneity was analyzed by SEM.

### 2.5 Scanning Electron Microscopy

Scanning electron microscopy (SEM) has been found to be the most appropriate and straightforward technique to gain insights into the inner structure of columns. In order to conduct optimization experiments, each individual capillary was marked with equally long sections, and each section was treated under different conditions with subcritical water (SCW). To assess the impact of individual parameters on the packing structure of the column and ensure reproducibility, the corresponding sections were cut into 5 pieces of around 5–10 mm in length using a ceramic knife. Thus, approximately 20–25 specimens (from 4 to 5 sections) were obtained from a single capillary, and perpendicular attachment to a supportive dural block was achieved using a specialized double-sided conductive adhesive tape. The dural block, along with the capillaries, underwent gold sputtering with a thin layer of gold (12–20 nm) using a Bal-tec/SCD 500 Sputter Coater through plasma discharge in a vacuum environment.

The gold sputtered samples were placed on a handling stage. They were then introduced under the tube of a Mira3 electron microscope (Tescan, Brno, Czech Republic). The microscope was operated at a voltage of 5 kV, with a working distance (WD) of 5 mm and beam intensity of 10 pA. The combination of secondary electrons (SE) and the InBeam detector was utilized.

### 2.6 MicroHPLC Instrument

The in-lab-assembled microHPLC setup for capillary column evaluation consisted of a syringe pump (100DM with d-series controller, Teledyne Isco, Lincoln, NE, USA), connected with an electrically actuated E90–220 injection valve with a 60 nL inner loop (Valco, Houston, TX, USA) and a passive T-splitter with a restrictor (fused silica capillary 25 μm i.d. × 150 mm length). The capillary column outlet was connected to a Spectra 100 UV–VIS detector (Thermo Separation Products, Waltham, MA, USA) via a fused silica capillary of dimensions 0.035 mm i.d. × 110 mm length with a bubble cell optical window (0.110 mm i.d.) made by controlled etching. The advantage of this arrangement is the low dispersion of analyte zone in the inlet capillary and a better detector response due to the dimensions of the bubble cell.

The actual flow rate through the column was continually monitored using Sensirion SLG64–0075 ultrahigh pressure flow sensor controlled by Sensor Viewer ver. 2.32 (Sensirion AG, Stäfa, Switzerland).

The chromatographic system was operated under constant pressure, because this arrangement gave more reproducible results. The flow rate was set indirectly by the pressure regulation.

All chromatographic measurements were carried out at laboratory temperature. UV-detection was performed at 210 nm. Data were collected and processed using DataApex Clarity 5.02 software (DataApex, Prague, Czech Republic).

## 3 Results and Discussion

### 3.1 Basic Considerations of Structures Formation

There has been sufficient theoretical and experiment-oriented literature on the phenomenon of liquid or solid bridge formation between particles or between a particle and a solid surface.^24−27^ Formation of these connections is not only important in many natural processes but also plays an irreplaceable role in many fields of industry. It is significantly involved in the saturation and retention of water in soil,^28−30^ it is extremely important in root surface-solid-particle-moisture interaction,^31^ and it has a significant effect on the adhesion of particles (dust, powder) to solid surfaces.^32−34^ In industry, it is then applied in the consolidation of granules and soil,^35,36^ in the formation of latex film,^37^ in the wetting of powders,^38,39^ and in the dispersion of pigments or in the production of antifoaming agents. These bridging forces have also been observed in atomic force microscopy,^40^ play a role in the attraction of two hydrophobic surfaces, etc. The above examples relate to moisture penetration into a material or matrix and in most cases involve the interaction of mutually insoluble liquid–solid phases.

**Figure 1:**
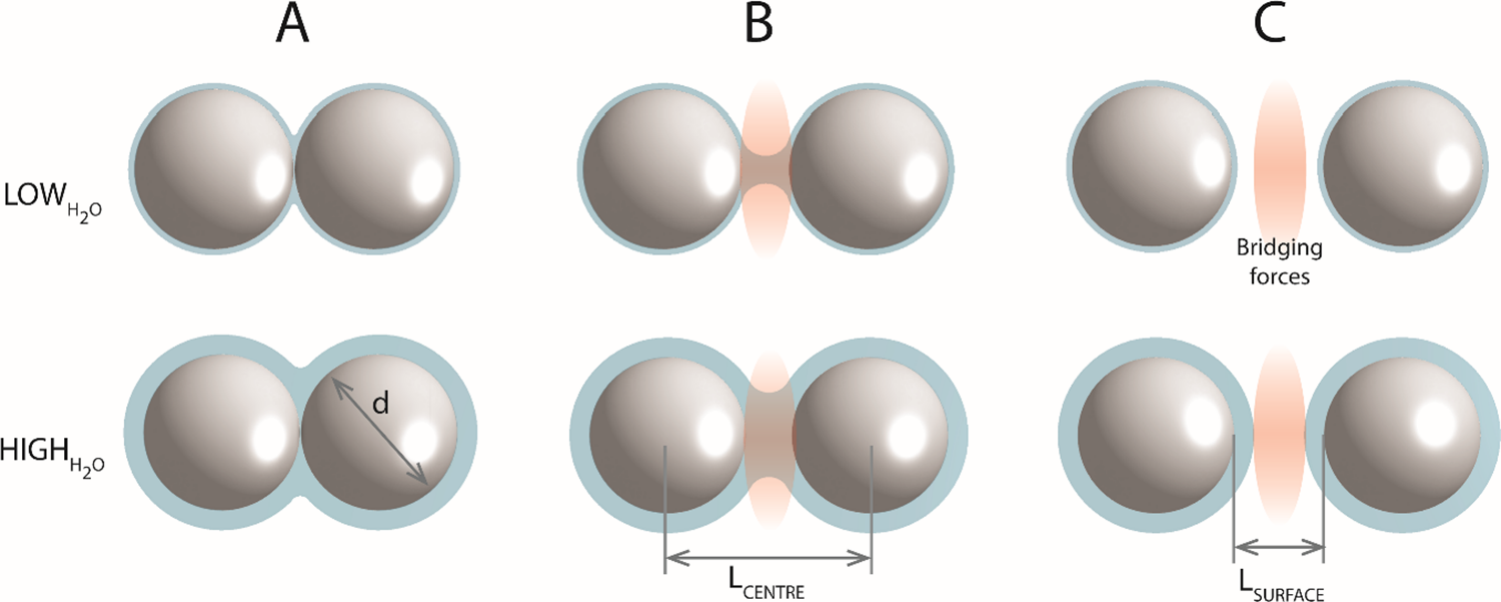
Wetting of differently spaced particles at low and high water content. (A) in contact, (B) bridge forming distance, (C) too far for forming bridge.

Simplistically, not mathematically, the process can be described in three cases (Figure 1): (A) when the particles pack in the closest possible way and the neighboring microspheres touch each other, *L*_surface_ = 0, (B) when the distance between the particle surfaces is small enough for the bridging forces to prevail, *L*_surface_ < *F*_bridge_, and (C) when the particle distance is so high that the formation of the connection does not occur at all, *L*_surface_ > *F*_bridge_. In natural processes, a combination of these three cases is expected to occur.

Figure 1 displays a demonstration of these three cases and the system’s behavior at low and high water levels. Depending on the level of moisture provided to the system, every particle forms a thinner or thicker surface layer of liquid and the corresponding size of the bridging, except in case C.

Regardless of the system configuration and water content, the geometric distance between the centers of the particles (Lcenter) and the shortest distance between the surfaces (Lsurface) remain constant. After drying, the system almost does not alter these geometric properties.

However, applying SCW to SiO_2_ particles changes the situation significantly. First, this is not a system of two immiscible phases as SiO_2_ has a certain level of solubility in SCW which can be controlled by the selection of the appropriate temperature and pressure. Second, the reverse process does not merely evaporate the water from the system. Instead, it recovers the dissolved SiO_2_ and converts the original liquid bridges into solid junctions.

**Figure 2:**
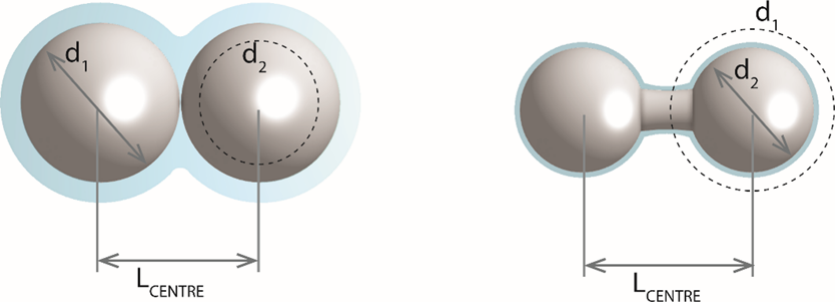
Formation of a bridge from material removed from particles.

Our opening assumption for the process occurring within a column filled with close-packed SiO_2_ microspheres, as depicted in Figure 1 type A and confirmed via SEM, is as follows:

When SCW is applied to the surface of the beads, a specific amount of SiO_2_ dissolves, forms a silicic acid gel layer, and reduces the diameter of the solid core of the beads from d_1_ to d_2_. This, in turn, creates a space between the surfaces of the beads *L*_surface_ > 0, which forms a liquid bridge subsequently. Depending on the selected parameters (time, temperature, pressure) and, in simple terms, based on the power of the solvent, the size of the gap and the resulting bridge may differ, core diameter is reduced from d_1_ to d_2_ while the distance between the centers *L*_center_ of the spheres remains constant (Figure 2).

**Figure 3:**
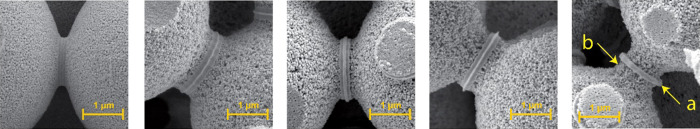
SEM images of the connecting bridges produced under different conditions with visibly different centers.

However, a series of experiments aimed at examining the formed junctions in detail did not entirely support these considerations but hinted at a somewhat distinct scenario instead. In fact, when the process proceeded according to the above assumption, the bridge formed from the recovered SiO_2_ would have to be materially homogeneous along its entire length. However, as demonstrated in Figure 3, even with experimental parameters chosen from a wide range, crystallographic homogeneity of the bridges was not guaranteed and the center of the bridge is visibly different.

**Figure 4:**
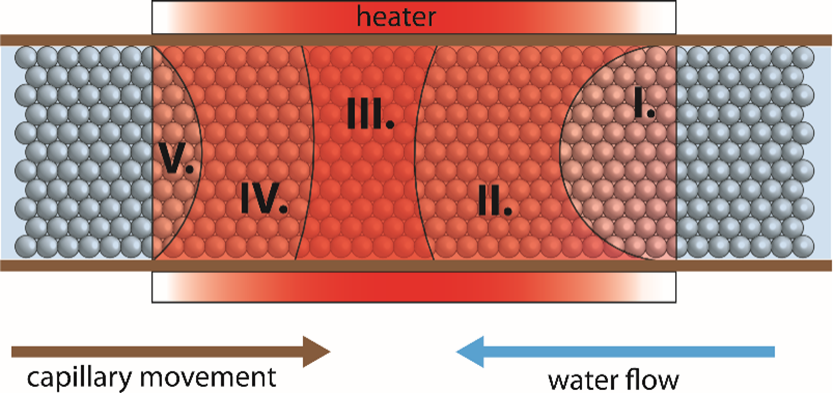
Schematic view of a possible sintering process. I. water preheating, II. etching of the formed 3D structure, III. liquid to solid bridge transformation, IV. liquid bridge formation, V. microspheres preheating.

Another indicator is the diameters of the bridges formed, which, contrary to expectations (Figure 1B), do not change much. It is therefore clear that the process has a different progression. As already mentioned, it is a fully dynamic process in which the capillary with the microspheres moves in the heated block, and the heated water flows counter currently through the capillary (Figure 4).

The regions I–V are schematically shown parts where the different steps of the sintering process occur at the steady state. The shape of the regions, their size, location, and perimeters cannot be determined in any simple way, also because these parameters change with the flow rate, the capillary movement speed, and the temperature. Since the pressure is nearly constant throughout the system and some degree of temperature gradient toward the outer edges of the heater can be assumed, a density gradient must be logically present in the system too.

**Figure 5:**
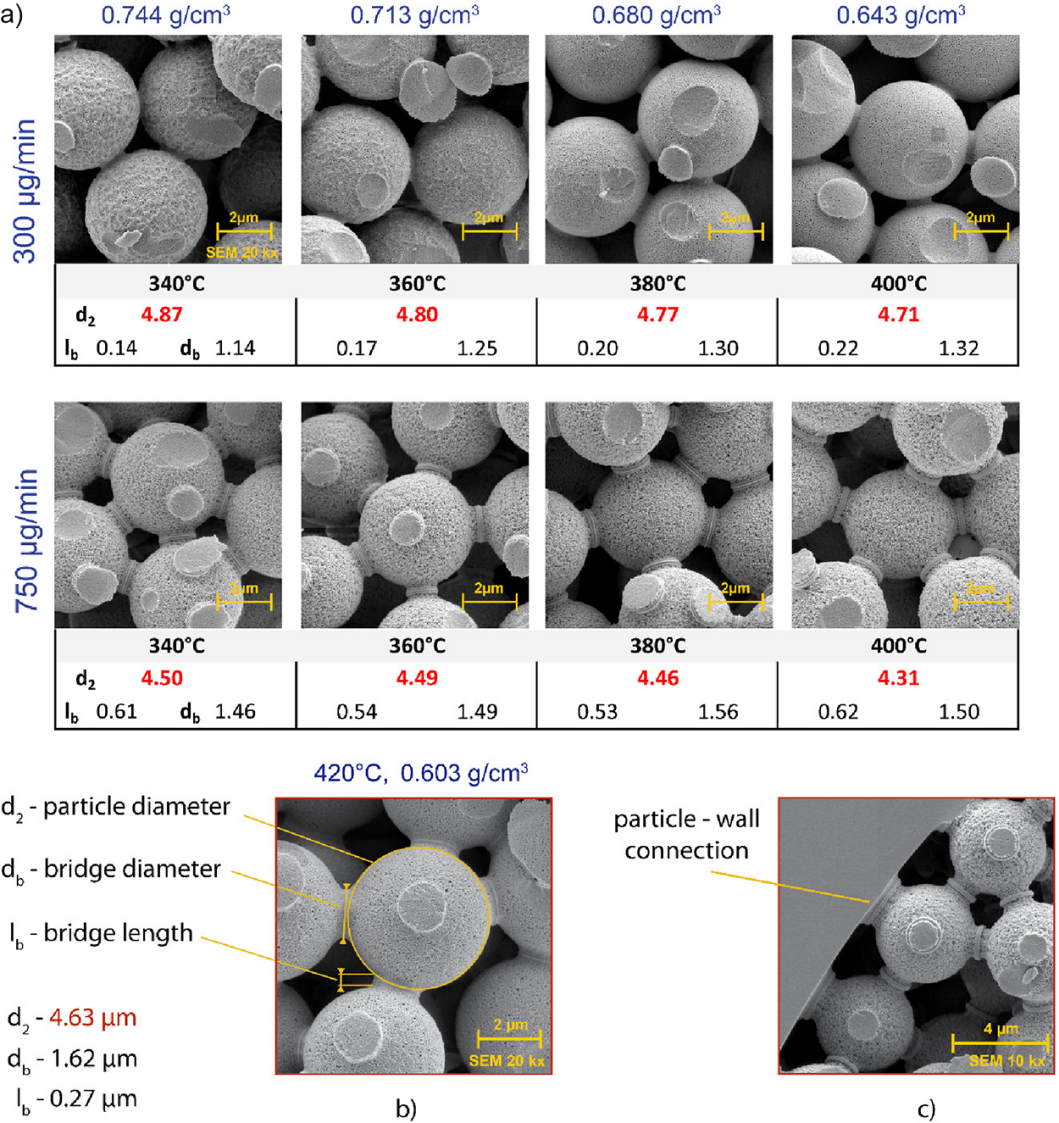
(a) SEM pictures of columns made at different temperatures. (b) Decrease in SiO_2_ solubility due to decrease in SCW density at high temperature. (c) Connection of microspheres to capillary wall.

This is essential for the process to proceed. Our simplified model assumes that in the first step, after entering the heater, the water and the microspheres are preheated in part I and part V, respectively. Since the movement of capillary with the microspheres is very slow, after heating in region V their temperature does not change much further until they enter region I, where they transfer some of the heat back to the incoming cool water. Even though the steps described below occur in the steady state simultaneously, let us imagine that the process starts in region IV, where the local water properties already allow the dissolution of a part of the microsphere surface. Thus, in this region, the maximum set temperature is not yet reached, but the actual temperature is already sufficient to form a silica gel film on the surface of the microspheres, and a liquid connection is formed between two particles or between a particle and the capillary wall (Figure 5c). The concentration of dissolved SiO_2_ at these bonds is many times higher than in the “free space” regions.^34^ As the beads move further into the hottest region III, where the set temperature is already reached, either a transition from subcritical to SCW or a significant decrease in water density occurs, depending on the conditions.

This results in a large decrease in the solubility of SiO_2_, its resolidification especially at high concentration points between microspheres and an overall decrease in the concentration of SiO_2_ in the SCW medium. The thus “weakened” medium regains the capacity to etch in region IV. The bonding thus formed is of a different crystallographic structure and is shown in Figure 3 by the homogeneous white ring (a). The whole 3D structure, already firmly bonded in this way, is moved to region II where it comes into contact with pure, unsaturated water at a temperature that allows the microsphere surface to be re-etched. Here, the SiO_2_ is already etched away from the formed 3D structure, the diameter of the bonded microspheres is reduced, while the solid bonds formed in the previous resolidification step are more or less resistant to this dissolution because of the different crystallographic structure. Since in all experiments the condition that linear velocity of H_2_O ≫ linear velocity of the capillary has been maintained, it is obvious that the steps are repeated many times before the capillary goes through the whole etching-sintering process.

This theory is confirmed by the fact that the fixed centers of the bridges retain a distance almost corresponding to the original diameter of the microsphere, i.e., to the points of original particle contact. The fact that the bridge's diameter d_b_ (Figure 5b) does not change much and are visibly formed by both recrystallization (Figure 3a) and etching (Figure 3b) also supports this theory.

### 3.2 Effect of SCW Temperature

The apparatus’s current design enables precise control over several parameters that directly impact the process, including temperature, pressure, capillary movement rate, and water flow-rate. The process can be run in either cocurrent or counter-current mode. At temperatures ranging from 300 to 500 °C (in 20 increments of 10 °C) and pressures ranging from 200 to 800 bar (in 30 increments of 20 bar), it is possible to perform 600 experiments while keeping all other parameters constant. Taking into account the influence of these parameters across the entire range mentioned, the number of possible experiments multiplies to tens of thousands. The preliminary measurements indicated that the resultant character of the 3D structure is most influenced by the SCW flow, temperature, and pressure. It is important to note that this evaluation is based on objective data and not subjective opinions.

In Figure 5a, SEM images of the column structures formed at a constant pressure of 725 bar and temperatures of 340–400 °C (0.744–0.643 g/cm^3^) are shown. It can be observed that at SCW flow rates of 300 μg/min and 340 °C, water is already able to dissolve the surface of SiO_2_ microspheres and the dissolved amount is even sufficient to form bonding bridges; however, the surface is highly heterogeneous due to only partial dissolving. Increasing the temperature to 360 °C increases the dissolving power of water, the amount of SiO_2_ etched increases but the microspheres still show residuals of the original surface. The use of 380 °C is already adequate for forming “hybrid microcolumns” as the microsphere surface is already uniform and the bridges are solid and consistent. Increasing the temperature to 400 °C confirmed the indicated trend and further increased the homogeneity of the formed structures. The reason why we use mass flow rate is that, unlike volumetric flow rate, it is invariant with respect to the water density changes resulting from the temperature and pressure changes needed for temporary and local generation of SCW inside the microsphere-packed capillary.

Figure 5a also shows the progress of an identical experiment performed at a flow rate of 750 μg/min, which confirms the trend described above, except that higher amounts of flowing water dissolve more SiO_2_, the process is much more intense and homogeneous columns can be prepared already at lower temperatures. As the temperature increases, the microsphere diameter decreases from the original 5.04 to 4.3 μm. This reduction in the diameter of the microspheres is directly related to the lengthening of the connecting bridges (*l*_b_), the increase in the free volume of the column, and the decrease in its hydrodynamic resistance. However, it is important to note that the process temperature cannot be increased indefinitely. As shown in Figure 5b, increasing from 400 °C (Figure 5a) to 420 °C at 750 μg/min, there is already a decrease in dissolution, and the resulting microsphere diameter is even larger than at 340 °C (4.63 vs 4.50 μm). In fact, at 420 °C and 725 bar, the density of SCW drops to 0.603 g/cm^3^ and SCW loses its dissolution ability. This indicates the existence of a minimum density, which requires the selection of a sufficiently high pressure to exceed, in addition to the temperature.

### 3.3 SCW Flow Rate Effect

The comparison of temperature dependencies measured at flow rates of 300 and 750 μg/min indicates a strong dependence on SCW flow. All experiments were carried out in counter-current mode with a constant capillary movement rate. This means that each microparticle of the capillary successively passes through the hypothetical regions (I–V) of the heated block and resides in each for a well-defined time. During this time, the material is exposed to SCW with properties defined by local pressure and temperature. Since the dissolving-sintering process does not only take place at a single specific point but is associated with the transport of dissolved SiO_2_, the whole process is significantly affected by the change in SCW flow rate.

First, as the SCW flow rate increases, each microsphere is repeatedly exposed to larger amount of water in the “dissolution” regions and thus more SiO_2_ is washed out of the system (microsphere diameter decreases); second, the size or shape of each region is likely to change due to heat transport.

However, despite the results of many experiments, this change need not be significant or the individual processes may be so fast that even with a change in flow rate from 200 to 1000 μg/min, consistent results are still obtained.

**Figure 6:**
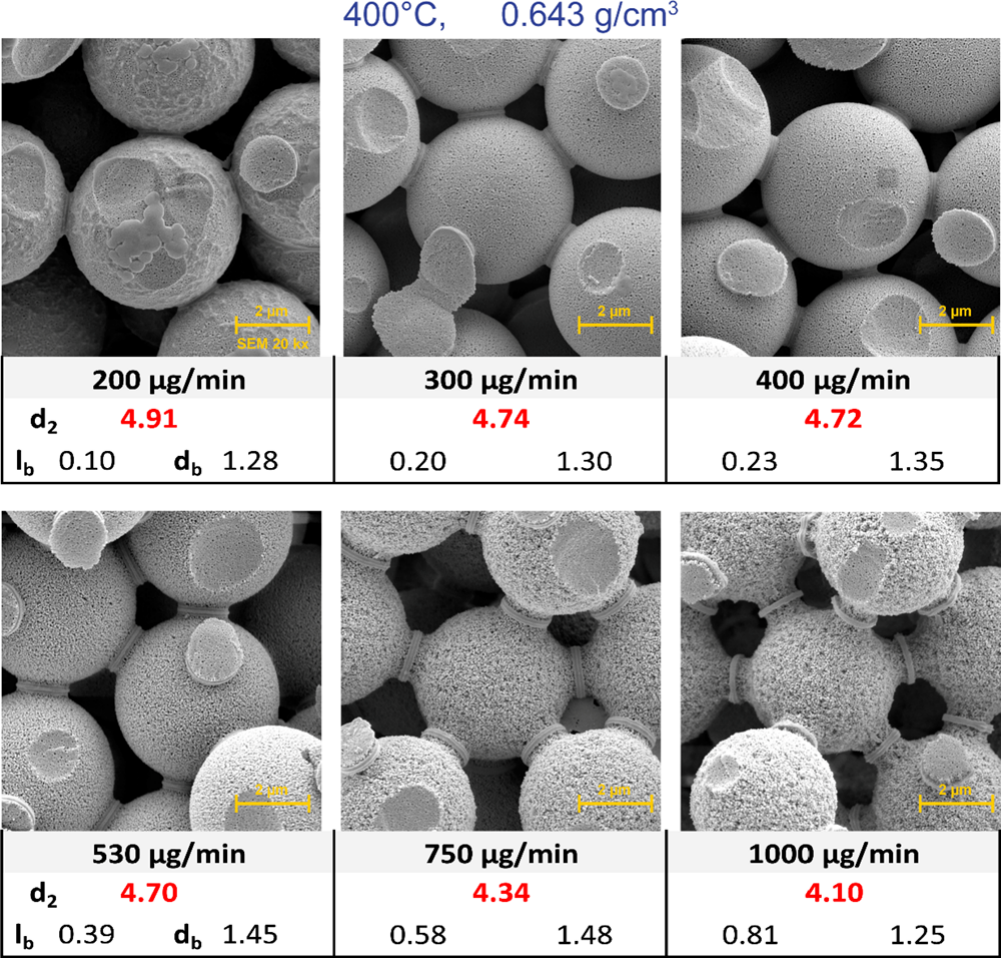
Effect of SCW flow rate change on column structures.

Figure 6 shows the 3D structures obtained at 400 °C, 725 bar (0.643 g/cm^3^), and at flow rates of 200, 300, 400, 530, 750, and 1000 μg/min. Except for 200 μg/min, where the amount of SCW supplied to the system is insufficient to completely dissolve the surface of the microspheres, all other structures produced are sufficiently homogeneous and suitable for use as a chromatographic column. Along with a noticeable reduction in microsphere diameter, a significant increase in “surface roughness” can also be observed. The formation of these micropores increases the overall surface area of the 3D structure and can play a positive role in both chromatographic and, for example, catalytic processes.

### 3.4 Reproducibility of Monolithic Column Preparation

The column preparation reproducibility investigations have included a (single) column homogeneity test and a column-to-column reproducibility test. The results of both tests are included in the Supporting Information file as Tables S3 and S4, respectively.

In the column homogeneity test, an SCW-treated column was cut into 1 cm long pieces, and sizes of microsphere diameter, bridge length, and bridge diameter were measured by SEM and compiled in Table S3. The 16 triplets of the resultant data thus cover the whole column length in equidistant proportions, and the individual parameter sets are characterized by the mean value, standard deviation, and relative standard deviation (RSD). The microsphere diameter shows the lowest RSD; this reflects a narrow size distribution of the starting silica microspheres as well as an apparently highly homogeneous etching/bridging process running along the whole column length. The bridge diameter still displays a fairly low RSD that probably reflects, at least in part, the quality of the packing procedure where the center positions of the individual microspheres remain fixed during the treatment with SCW; this feature, together with the uniform reduction in microsphere diameter, results in relatively narrow distribution of bridge lengths. The bridge diameter appears to have the highest RSD, in fact not surprisingly. Of the three geometric parameters, this one is probably the least significant. The most important point, however, is that none of the three geometric parameters shows any definite trend along the column length, testifying to a homogeneous etching/bridging process.

Table S4 shows the statistics data pertaining to the monolith fabrication reproducibility test (column-to-column reproducibility). Most RSDs in Table S4 are more favorable than those in Table S3 despite the lower number of data points. The probable explanation is that the operating conditions used to acquire the data in Table S4 were different from those in Table S3 (please see the footnotes to the respective photograph sets in the Supporting Information file). Table S4 also includes the “aggregated” column-to-column statistics indicating very good column-to-column reproducibility of the monolith fabrication via the SCW-induced etching/bridging procedure.

### 3.5 Characterization of Synthesized Material by Liquid Chromatography

**Figure 7:**
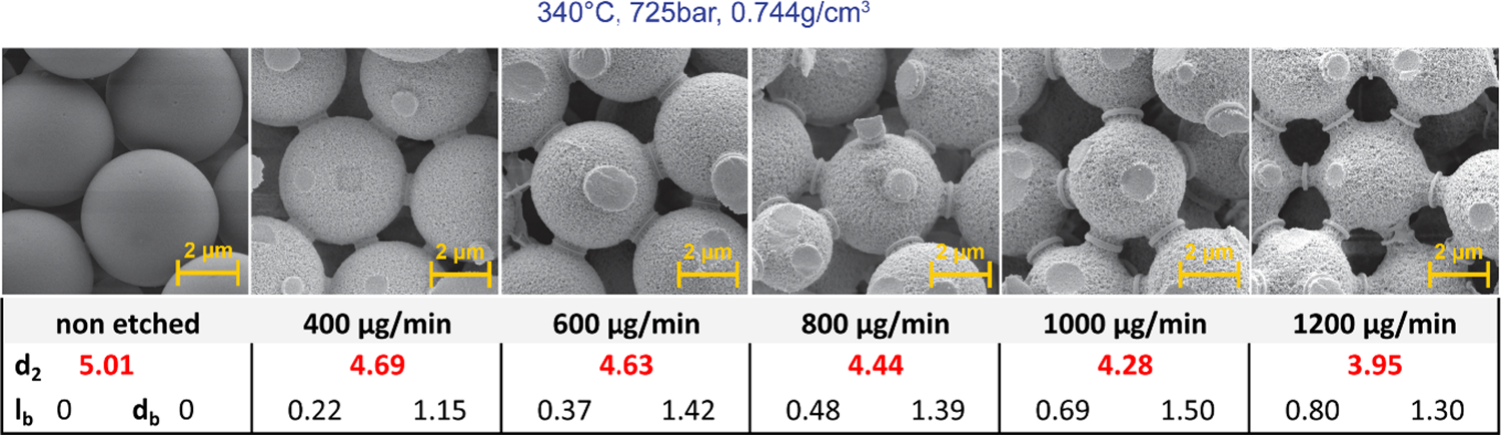
Comparison of columns treated with SCW with a nonetched column.

Based on the results of previous experiments, the operating conditions were selected and five chromatographic columns with homogeneous 3D structures were fabricated. For the columns, the microsphere size was gradually reduced using SCW while increasing the column permeability. The geometrical characteristics of each column together with SEM images are shown in Figure 7.

It should be emphasized here that, because of the introductory character of this study, most experiments in this section were performed with monolithic columns just after the SCW treatment. The only exception was the example separation of a mixture of alkylbenzenes where, after the SCW treatment, the column was chemically modified with ODS to produce a C18 stationary phase. After visual observation by SEM, characterization of silica monolithic material by liquid chromatography was performed.

The prepared capillary columns have a very low volume. Typical length of the column with i.d. 100 μm was 150 mm, which corresponds to volume 1.2 μL for an empty capillary; this value is further reduced by the volume of the particles present. Therefore, to obtain relevant results, it was important to minimize dead volumes throughout the chromatographic system.

Because of the injector contribution to zone broadening, the splitter (restrictor capillary i.d. Twenty-five or 30 μm, splitting ratio about 1:50) was inserted between the sampling valve and the column. This improved the shape of the injected zone significantly. Another source of zone broadening was the capillary optical cell in the detector. As the peak variance in an open capillary depends on the sixth power of the radius (*r*^6^),^41^ it is necessary to minimize especially the diameter of the capillary. However, detection sensitivity depends on the first power of the capillary diameter (*r*). As a good compromise, we prepared an etched bubble optical cell of 110 μm i.d. in a fused silica capillary of 35 μm i.d.

Before the measurements, the end faces of the prepared columns were ground and polished on sandpapers (1000, 2500) in the preparer to be smooth and perpendicular to the column axis. In what follows, the important chromatographic characteristics^42^ of the individual columns are discussed and compared with those of an SCW-untreated capillary column packed with discrete silica microspheres.

#### 3.5.1 Column Efficiency

The column efficiency is a measure of the width of the elution zone when it passes through the detector. In an ideal case, these bands have a Gaussian concentration profile. The narrower the zone, the more efficient the column. The plate number (*N*) expresses the efficiency of the column and it can be calculated as
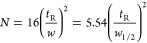
where *t*_R_ is the retention time of the peak maximum and *w* is the baseline width. It is more accurate to determine the width of the peak at half of its height, *w*_1/2_. Number of theoretical plates *N* is usually expressed per meter of the column, N/m. Another way of expressing chromatographic efficiency is by moment analysis.^43^

We measured efficiency in the acetonitrile/water (90/10) mobile phase by injection of toluene solution (1 μL/mL in the mobile phase). In this chromatographic system, toluene is not retained on the surface of SiO_2_ and elutes approximately in the void time (*t*_0_). We set the flow rate of the mobile phase to have linear velocity in the range of 1.0–1.5 mm/s, which is optimal for 5 μm particles.^20^

From Table 1, it is obvious that the SCW treatment affected all etched columns and increased N/m. Untreated particles provide about 85,000 plates/m which is a typical value for a column packed with 5 μm sorbent, and we obtained similar values repeatedly for all untreated columns.

**Table 1:** Measured Chromatographic Parameters (Left Side), Average Values *n* = 3 (Right Side)

SCW flow	length [mm]	dp [MPa]	N/m W_50%_^a^	N/m stat.mom.^b^	ε total	*k* [× 10^–14^m^2^]	*E*	N/m W_50%_^a^	N/m stat.mom.^b^	ε total	*k* [× 10^–14^m^2^]	*E*
nonetched	153	3.1	96,000	96,000	0.313	1.13	9581	86,667	97,333	0.313	1.13	11931
	153	3.1	82,000	98,000	0.312	1.13	13,132					
	153	3.1	82,000	98,000	0.315	1.14	13,080					
400 μg/min	155	2.0	112,000	134,000	0.358	2.49	3205	116,667	128,000	0.360	2.51	2955
	155	2.0	113,000	125,000	0.364	2.51	3123					
	155	2.0	125,000	125,000	0.358	2.52	2538					
600 μg/min	155	1.3	137,000	173,000	0.394	2.91	1834	137,333	147,000	0.390	2.88	1840
	155	1.3	136,000	135,000	0.385	2.87	1883					
	155	1.3	139,000	133,000	0.390	2.87	1803					
800 μg/min	152	1.5	1,325,001	160,000	0.468	3.39	1679	133,500	146,000	0.454	3.32	1694
	152	1.5	138,000	138,000	0.454	3.28	1599					
	152	1.5	130,000	140,000	0.439	3.28	1802					
1000 μg/min	154	0.8	113,000	118,000	0.509	5.90	1327	114,333	125,333	0.509	5.87	1303
	154	0.8	115,000	128,000	0.509	5.87	1287					
	154	0.8	115,000	130,000	0.508	5.84	1294					
1200 μg/min	145	1.0	117,000	156,000	0.563	6.76	1080	121,333	141,333	0.563	6.88	990
	145	1.0	123,000	125,000	0.562	6.94	953					
	145	1.0	124,000	143,000	0.564	6.94	937					

a Calculated assuming Gaussian concentration profile.

b Calculated from statistical moments by Data Apex clarity 5.02 software.

After the SCW treatment, values of N/m increased in all cases up to a maximal value of about 139,000 plates/m; this number corresponds to the efficiency of a column packed by silica microspheres of about 3 μm size. It appears that the treatment has some optimum between 600 and 800 μg/min of the SCW flow. The important fact is that this excellent efficiency was achieved at a 2.5× smaller pressure drop when compared with untreated microspheres.

**Figure 8:**
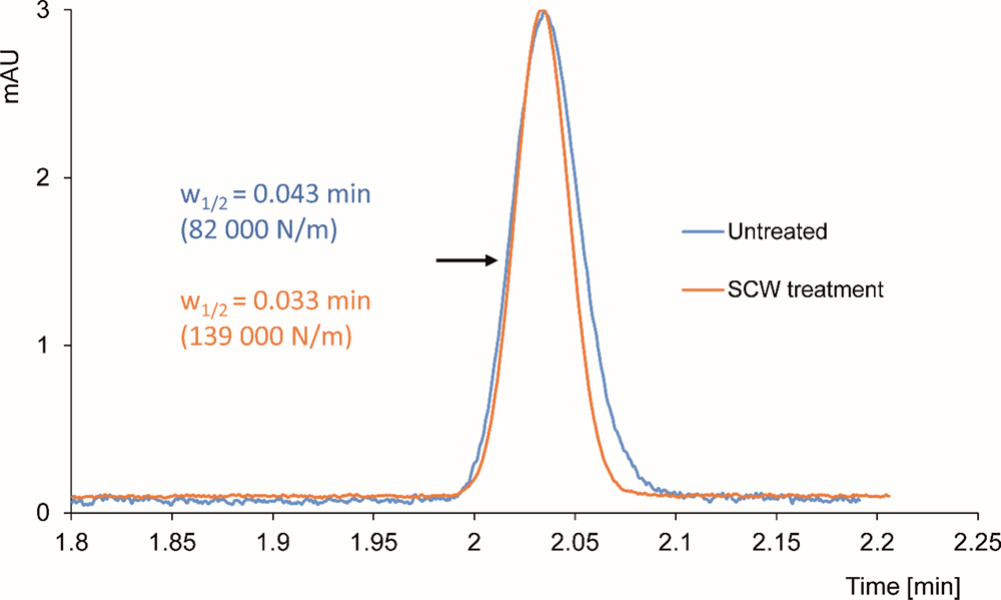
Peak profiles of unretained compound (toluene), eluted from chromatographic columns packed by untreated (blue), or SCW treated (red) silica microspheres. The flow rate of the mobile phase (90% ACN) was set in both cases to obtain equivalent elution times, and the height of peaks was adjusted to the same level for profile comparison.

Figure 8 shows the effect of SCW treatment (800 μg/min) on the zone profile as eluted from the column.

Because of different total porosities of the columns (0.313 for the untreated one and 0.454 for the SCW-treated one), the amount of the mobile phase in each column was different, and the difference resulted in different final flow rates (246 and 328 nL/min, respectively) to obtain the same retention times. In accordance with the discussion above, the SCW treatment results in a marked narrowing of the eluted zone. We observed a change of efficiency from 82,000 to 139,000 plates/m when measured by the width of the peak at half of its height. When efficiencies were calculated from statistical moments (by DataApex Clarity 5.02 software), values of 98,000 for the untreated column and 160,000 for the SCW-treated column were obtained.

Measurements with little retained substance in the system (toluene) gave only indicative information about the effect of SCW treatment on efficiency. In this case, there are minimal interactions between the phases and the analyte, and the resulting efficiency mainly reflects the homogeneity of the column bed. The next step was to investigate the analytes with a higher retention. For this purpose, one of the SCW-treated columns (800 μg/min) and one column packed with untreated particles were submitted to ODS modifications (described in Section 2.2).

**Figure 9:**
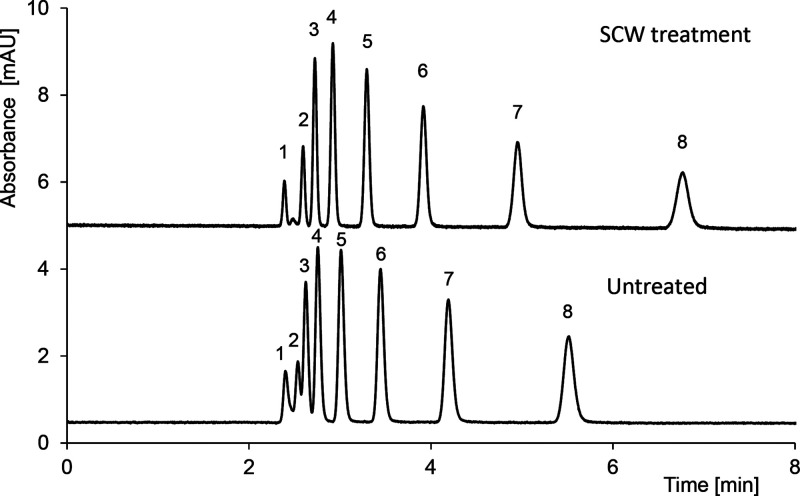
Chromatographic evaluation of SCW treated (upper) and untreated (bottom) columns, both modified with ODS to produce the C18 stationary phase. Mobile phase: 50% acetonitrile/water. Tested compounds: (1) uracil, (2) benzene, (3) toluene, (4) ethylbenzene, (5) propylbenzene, (6) butylbenzene, (7) pentylbenzene, (8) hexylbenzene. Data of the chromatograms are in the Supporting Information file Table S1 (upper); Table S2 (lower).

Figure 9 shows the influence of SCW treatment to separation of uracil + alkylbenzenes in 50% acetonitrile on C18 modified silica columns. For the SCW-treated column, the performance for uracil (as a practically unretained analyte) and benzene (as a compound with low retention) confirmed values (130,000 plates/m), which we obtained for toluene (in 90% ACN). By comparing Tables S1 and S2, it is possible to determine the effect of treatment on the spectrum of analytes with higher retention factors.

The highest number of theoretical plates reached for both columns corresponds to the values obtained earlier—about 80,000 for the untreated column and 130,000 for the SCW-treated column (see Supporting Information, Tables S1 and S2).

The SCW treatment process significantly enlarges the surface area of the particles. As can be seen from Figure 7, SCW-treated particles have visibly rougher surfaces, which provides a larger surface area. With subsequent ODS modification, more stationary phase is anchored on the surface, resulting in higher retention of the separated analytes.

#### 3.5.2 Total Porosity of the Columns

The total porosity of a column ε_T_ is the fraction of the bed volume which is occupied by the mobile phase. It is a dimensionless parameter ranging between 0 for no void space and 1 for an open tubular column. In the chromatographic system used for the present measurement, the volume of the capillary connecting the column outlet with the UV detector cell cannot be neglected with respect to the total mobile phase volume in the column. Total porosity was calculated from
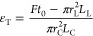
where *F* is the volumetric flow rate of the mobile phase, *t*_0_ is the void retention time of toluene (for nonporous particles), *r*_L_ and *L*_L_ are the inner radius and the length of the connecting capillary of the optical cell, respectively, and *r*_C_ and *L*_C_ are the inner radius and the length of the column, respectively.

In Table 1, total porosities for all columns are presented. Nonetched particles exhibited the value (0.313) which is near to the ideal value for close-packing of equal spheres (≈0.26). After the SCW treatments, we observed a significant increase in ε_T_ values from 0.360 to 0.563. It indicates a marked decrease of SiO_2_ volume in the column which was also confirmed by the SEM observations in Figure 7.

#### 3.5.3 Column Permeability

The term permeability describes how easily the liquid (mobile phase) flows through the column packed with a stationary phase (particles or monolith). At a given pressure, the higher is flow through the column the higher the permeability of the column. A more exact definition of permeability is described as the volume flow of fluid per unit time per unit area per unit pressure gradient. The column permeability can be easily calculated from the flow and column characteristics using the following equation:
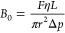
Here, *F* is the mobile-phase flow rate, η is the mobile-phase viscosity, Δ*p* is the pressure drop across the column, *L* is the column length, and *r* is the column inner radius.

From Table 1, it can be seen that the permeability *B*_0_ of particles after treatment grows linearly with the SCW flow rate. When compared with nonetched particles, we can observe more than a 6× increase in column permeability. From a chromatographic point of view, higher permeability of the column is preferred because it reduces the pressure needed in the system. In addition, a more viscous mobile phase can be used for separation.

#### 3.5.4 Separation Impedance

The separation impedance *E* represents the difficulty of achieving a certain performance and should be minimized for optimum performance. The highest performance is achieved by a column which combines low resistance to flow and minimum dispersion of chromatographic solute bands. For an unretained compound, *E* is given^42^ by
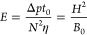


The calculated separation impedances for SCW-treated and nontreated columns are listed in Table 1. As can be seen, even the smallest SCW flow rate dramatically decreased the value of *E*. In other words, the overall column performance is better when a higher SCW flow rate is used for the treatment. The first, nontreated column has an *E* of almost 12,000. For a good separation impedance *E* should be lower than 10,000.^44^ Thus, this column performance is reduced. The gradual increase in SCW flow rate leads to a significant decrease of the separation impedance and for final column *E* is more than 12× lower.

Overall, the data listed in Table 1 illustrate the power of the SCW-assisted route to a monolithic column from a column packed with discrete silica microspheres. It turns out that, even when using just a very limited introductory set of operating conditions, the efficiency, total porosity, permeability, and separation impedance of the resulting column can be adjusted within rather wide limits.

## 4 Conclusions

This pilot work presents a new and unique method for preparing 3D homogeneous structures using SCW’s ability to dissolve silica. The method’s high variability is due to the wide range of applicable temperatures and pressures that can be used to tune the properties of SCW, among other parameters. The likely course of the process is also described. Chromatographic columns prepared using this method exhibit high separation efficiency while maintaining high permeability. The microspheres are not only connected to each other but also to the capillary wall, resulting in a rigid cartridge that is free from any movement. Therefore, unlike conventional packed columns, this column does not require frits at its ends, it can be shortened at will if necessary, and the mobile phase can flow through the column in either direction.

Although there are several limitations of this introductory study, namely, the absence of pore size measurements and the absence of surface characterization employing the inverse size exclusion chromatography, feasibility of the technique has been clearly indicated and a possible mechanism of 3D structure formation has been suggested. As regards the mechanical stability and durability of the 3D framework, no flow fluctuations indicative of 3D structure collapse were observed during the column testing. In addition, we did not observe any decrease in column efficiency (even after several drying/moistening cycles), which itself is a good indicator of 3D structure mechanical stability. The chemical stability of the synthesized material is assumed to be the same as for unmodified silica sorbents, i.e., stability up to pH = 8. In our experimental experience, the high temperature alone causes neither structural collapse nor damage to silica microspheres. On the contrary, the high temperature is necessary for bringing local water into near- or supercritical state which is needed for solubilizing silica and forming the connecting bridges between nearest neighbor microspheres (or between a microsphere and the inner wall of the fused silica capillary). Depending on the conditions, there is a significant reduction in particle diameter from 5.04 to 3.95 μm, resulting in a decrease in hydrodynamic resistance. This effect would be more pronounced for smaller original microspheres. Currently, high-efficiency microcolumns with close-packed microsphere sizes of 3 and 1.7 μm require the use of very high pressures of 700–1000 bar (ultra high-pressure liquid chromatography) because of their high hydrodynamic resistance. However, with the new method, this limitation can be effectively curbed or even completely eliminated. The use of pure water throughout the process and the absence of heteroatoms on the surface of the structure can be crucial in biochemical analyses.

In order to assess the performance of the new type of monolithic columns, an SCW-treated column and a discrete microsphere-packed column were both chemically modified to introduce the C18 stationary phase. A simple comparison using a mixture of alkylbenzenes indicated superior performance of the SCW-treated, bridged-microsphere column over the discrete microsphere-packed column.

## Supporting Information Available

Flowchart of the three-step procedure (CO_2_ packing, SCW treatment, chemical modification) to treat the packed capillary column with sub/supercritical water; chromatographic performance data of SCW-treated and SCW-untreated columns, both modified with ODS to produce the C18 stationary phase; fabricated column in overall view (the whole cross-section), at medium and high magnification; test of monolith homogeneity; test of column-to-column reproducibility; measured sizes and statistics data; measurements of the source particle distribution (PDF)

## Abbreviations

SCW: supercritical water
PHW: subcritical water
FSC: fused silica capillary.
