# Supplementary material for: Forming Homogeneous Three-Dimensional Structures from Discrete Silica Microspheres Using Sub/Supercritical Water
Source: ACS Appl Mater Interfaces. 2024 Aug 1;16(32):42873–83. doi: 10.1021/acsami.4c07251 (PMC11331445; doi:10.1021/acsami.4c07251)
Supplement: Supplementary file 1 — am4c07251_si_001.pdf [file am4c07251_si_001.pdf]

Supporting Information to

Manuscript ID am-2024-07251a

Forming Homogeneous Three-Dimensional Structures from Discrete Silica Microspheres Using Sub/Supercritical Water

Pavel Karásek,[*] *Josef Planeta, Michal Roth*

Institute of Analytical Chemistry of the Czech Academy of Sciences, Veveří 97, 60200 Brno, Czech Republic

Contents:

- Flowchart of the procedure for treating the packed capillary column with sub/supercritical water
- Tables comparing the chromatographic performance data of SCW-treated and SCW-untreated columns, both modified with ODS to produce C18 stationary phase
- Example of randomly selected images showing the fabricated column in overall view (the whole cross-section), at medium and high magnification.
- Test of monolith homogeneity
- Test of column–to–column reproducibility
- Tables S3 and S4 showing measured sizes and statistics data
- Measurement of the source particles distribution

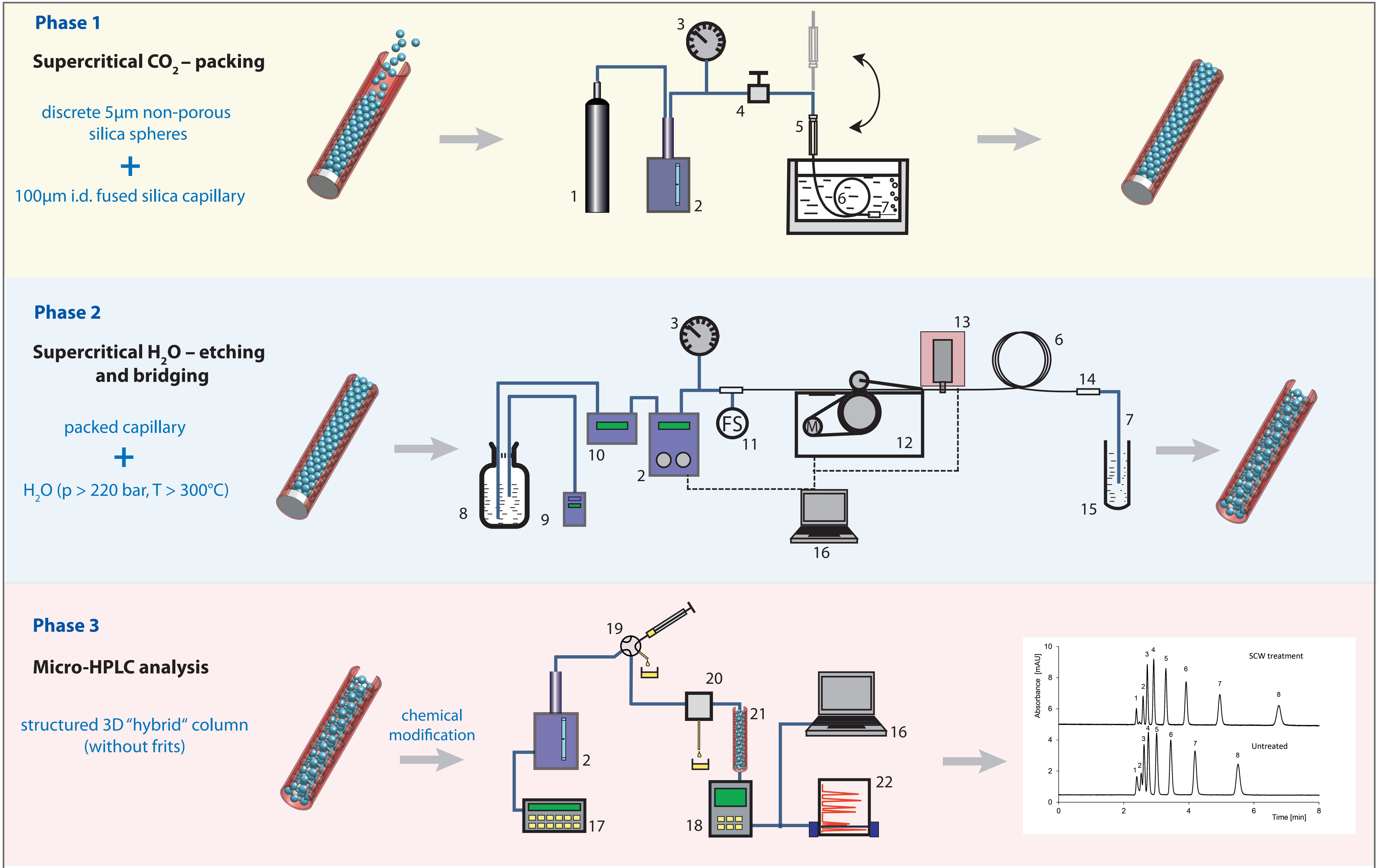

1 - CO_2 cylinder, 2 - high pressure pump, 3 - pressure sensor, 4 - on-off valve, 5 - filling reservoir, 6 - packed FS capillary, 7 - restrictor, 8 - H_2O reservoir, 9 - oxymeter, 10 - degasser, 11 - flowmeter, 12 - programmable moving device, 13 - heater (SCW generator), 14 - high pressure coupling, 15 - waste, 16 - process control and data collection, 17 - pump control, 18 - UV/VIS detector, 19 - computer controlled injection valve, 20 - splitter, 21 - "hybrid" column, 22 - data collection

Table S1: Chromatographic separation of alkylbenzenes on SCW-treated column and ODS-modified column

Peak	Reten. time [min]	W05 [min]	Asymmetry [-]	Capacity [-]	Efficiency [th.pl]	Eff/l [t.p./m]	Resolution [-]	Compound Name
1	2.390	0.040	0.909	0.00	19833	**130483**		Uracil
2	2.593	0.043	1.182	0.08	19893	**130874**	2.879	Benzene
3	2.722	0.050	1.167	0.14	16475	**108390**	1.644	Toluene
4	2.922	0.053	1.000	0.22	16682	**109753**	2.284	Ethylbenzene
5	3.295	0.063	1.059	0.38	15041	**98953**	3.776	Propylbenzene
6	3.914	0.077	1.150	0.64	14483	**95285**	5.226	Butylbenzene
7	4.952	0.100	1.071	1.07	13629	**89666**	6.946	Pentylbenzene
8	6.766	0.146	1.075	1.83	11827	**77810**	8.691	Hexylbenzene

Column: i.d. = 0.1 mm, length = 152 mm, stationary phase: C18, mobile phase: 50% acetonitrile / water

Table S2: Chromatographic separation of alkylbenzenes on SCW-untreated column and ODS-modified column

Peak	Reten. time [min]	W05 [min]	Asymmetry [-]	Capacity [-]	Efficiency [th.pl]	Eff/l [t.p./m]	Resolution [-]	Compound Name
1	2.400	0.057	1.385	0.00	9937	**62109**		Uracil
2	2.537	0.053	0.917	0.06	12533	**78328**	1.466	Benzene
3	2.623	0.057	1.067	0.09	11873	**74206**	0.930	Toluene
4	2.757	0.063	1.200	0.15	10496	**65598**	1.311	Ethylbenzene
5	3.010	0.070	1.294	0.25	10243	**64022**	2.242	Propylbenzene
6	3.447	0.080	1.200	0.44	10283	**64270**	3.485	Butylbenzene
7	4.190	0.097	1.250	0.75	10408	**65053**	4.965	Pentylbenzene
8	5.517	0.127	1.088	1.30	10508	**65678**	7.010	Hexylbenzene

Column: i.d. = 0.1 mm, length = 160 mm, stationary phase: C18, mobile phase: 50% acetonitrile / water

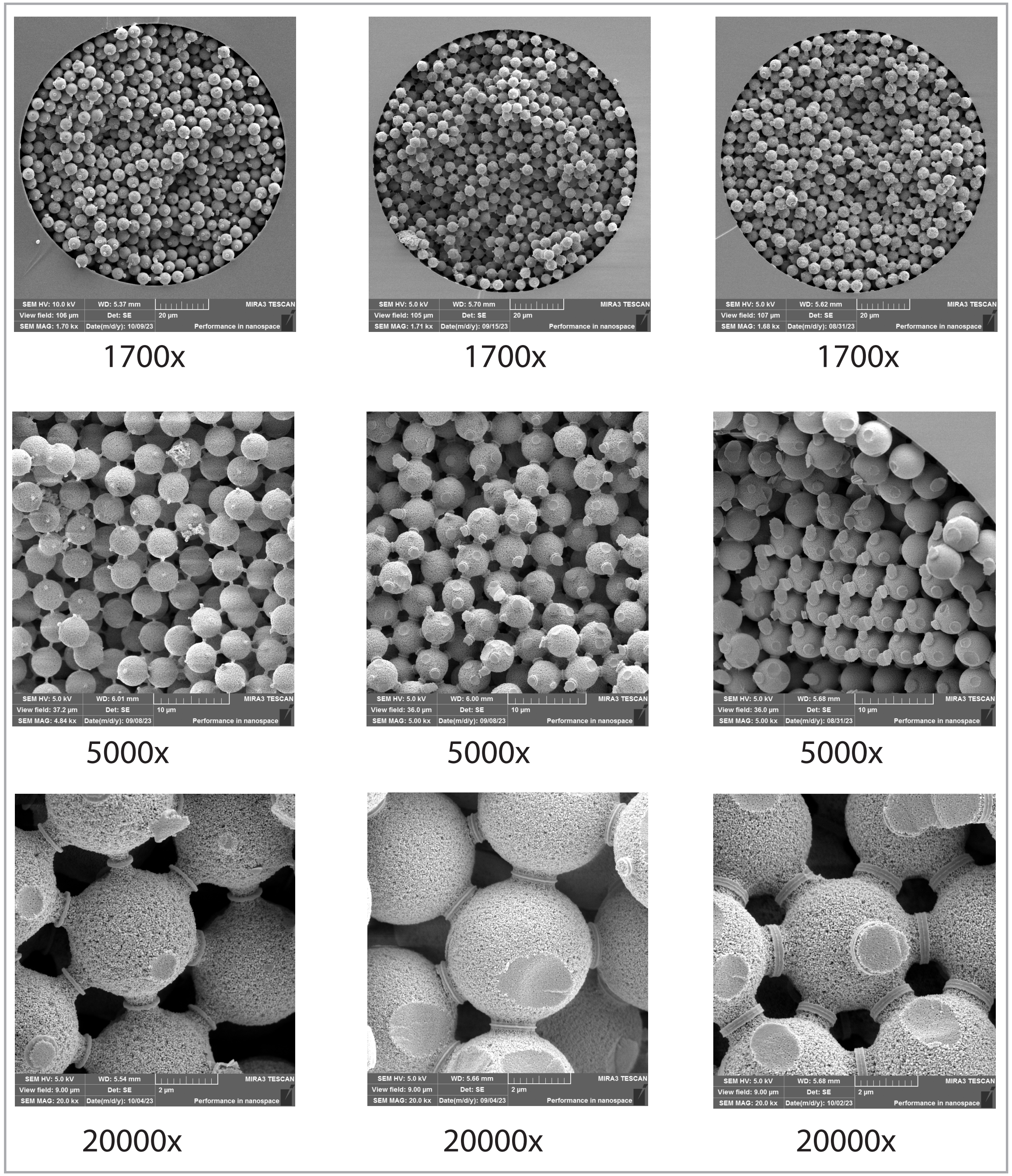

Example of randomly selected images showing the fabricated column in overall view, at medium and high magnification. A magnification of 20000x was chosen in the manuscript to allow better visibility of surface detail and for more accurate measurement of geometric parameters.

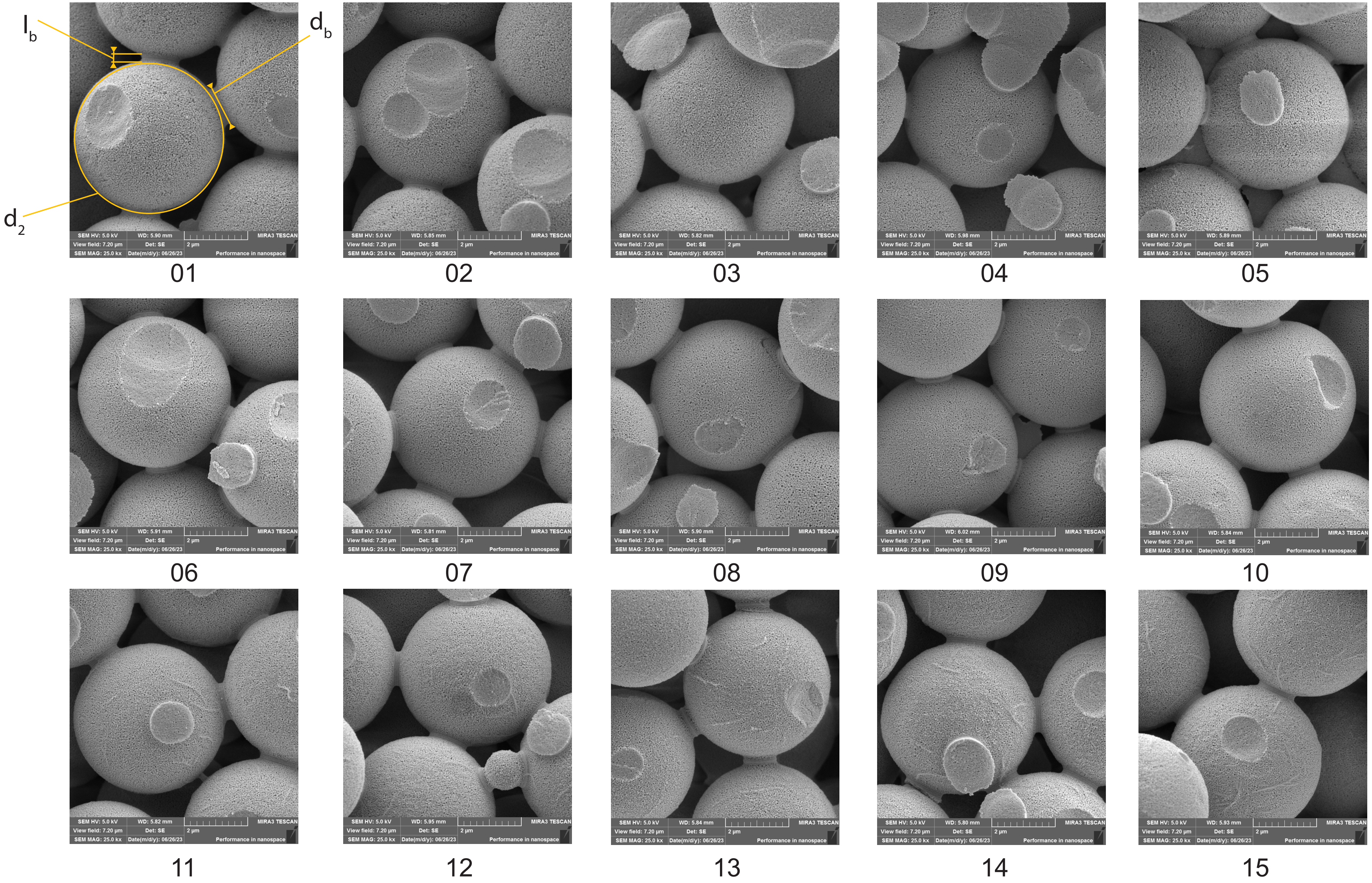

Test of monolith homogeneity. The column was cut into 1cm pieces and analyzed by SEM at 25000x magnification. Size measurements results and statistics are enclosed in Table S3.

Conditions: pressure 700 bar, temperature 370° C, SCW flowrate 400 µg/min, linear moving velocity 1.18 mm/min.

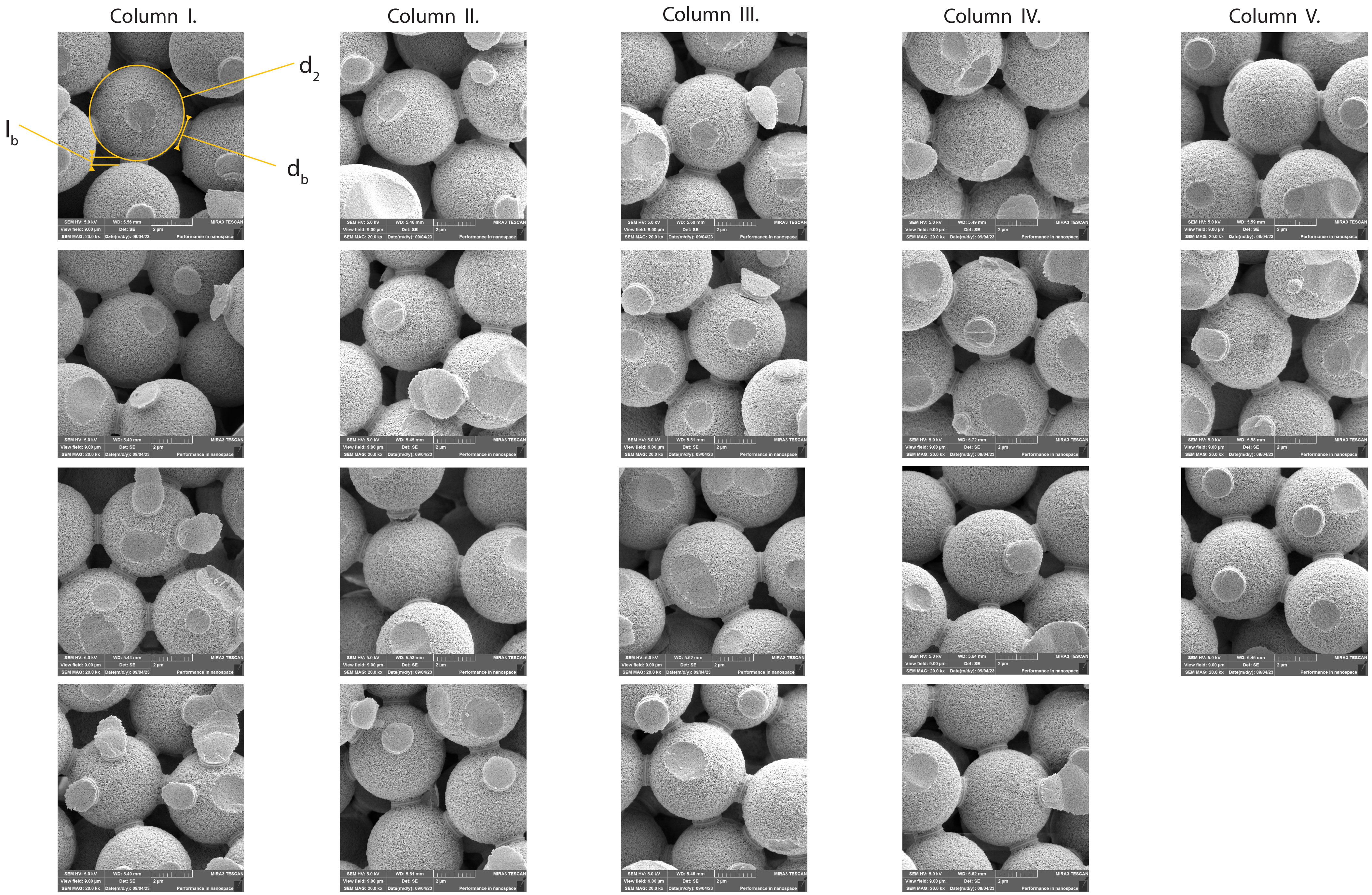

Test of column-to-column reproducibility at monolith process fabrication. The five columns were cut into several equal lengths and analysed by SEM at 20000x magnification. Size measurements results and statistics are enclosed in Table S4.
Conditions: pressure 725 bar, temperature 340 °C, SCW flowrate 500 µg/min, linear moving velocity 0.79 mm/min.

Table S3: Test of monolith homogeneity, statistics data

Cut No.	spheres diameter d_2 [µm]	bridge length l_b [µm]	bridge diameter d_b [µm]
1	4.760	1.228	0.213
2	4.764	1.256	0.217
3	4.808	1.280	0.205
4	4.748	1.279	0.219
5	4.742	1.198	0.204
6	4.800	1.260	0.203
7	4.760	1.215	0.202
8	4.780	1.265	0.206
9	4.782	1.192	0.196
10	4.860	1.225	0.204
11	4.756	1.222	0.183
12	4.798	1.228	0.160
13	4.648	1.078	0.292
14	4.824	1.199	0.170
15	4.822	1.241	0.163
16	4.818	1.246	0.164
**Mean [µm]**	**4.779**	**1.226**	**0.200**
**SD [µm]**	**0.048**	**0.048**	**0.032**
**RSD [%]**	**1.005**	**3.921**	**15.732**

Table S4: Test of monolith fabrication reproducibility, statistics data

Column No._cut	spheres diameter d_2 [µm]	bridge length l_b [µm]	bridge diameter d_b [µm]
I_1	4.660	1.390	0.310
I_2	4.680	1.380	0.330
I_3	4.700	1.490	0.380
I_4	4.600	1.450	0.320
II_1	4.700	1.480	0.330
II_2	4.760	1.490	0.310
II_3	4.680	1.470	0.315
II_4	4.660	1.510	0.320
III_1	4.660	1.500	0.340
III_2	4.700	1.490	0.360
III_3	4.740	1.510	0.310
III_4	4.740	1.480	0.350
IV_1	4.660	1.490	0.340
IV_2	4.756	1.512	0.307
IV_3	4.686	1.500	0.302
IV_4	4.680	1.500	0.306
V_1	4.760	1.480	0.360
V_2	4.660	1.490	0.320
V_3	4.680	1.520	0.330

Column No.		spheres diameter d_2 [µm]	bridge length l_b [µm]	bridge diameter d_b [µm]
I	**Average [µm]**	4.660	1.428	0.335
	**SD [µm]**	0.043	0.052	0.031
	**RSD [%]**	0.927	3.634	9.281
II	**Average [µm]**	4.700	1.488	0.319
	**SD [µm]**	0.043	0.017	0.009
	**RSD [%]**	0.920	1.148	2.679
III	**Average [µm]**	4.710	1.495	0.340
	**SD [µm]**	0.039	0.013	0.022
	**RSD [%]**	0.813	0.864	6.354
IV	**Average [µm]**	4.700	1.501	0.314
	**SD [µm]**	0.042	0.009	0.018
	**RSD [%]**	0.891	0.600	5.620
V	**Average [µm]**	4.700	1.497	0.337
	**SD [µm]**	0.053	0.021	0.021
	**RSD [%]**	1.126	1.391	6.183
**column-to-column**	**Average [µm]**	**4.693**	**1.481**	**0.329**
	**SD [µm]**	**0.019**	**0.031**	**0.012**
	**RSD [%]**	**0.410**	**2.060**	**3.577**

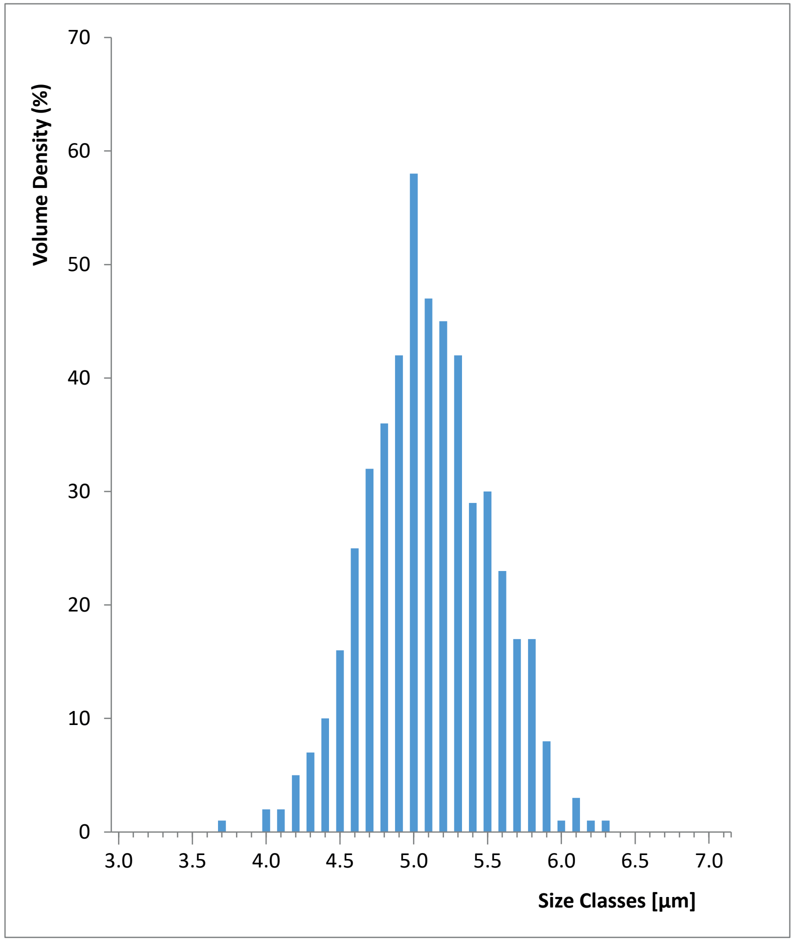

Distribution of source particle sizes. Data obtained from particles manufacturer: mean 5.04µm, deviation 0.408 µm.

The histogram on the left shows the results obtained in laboratory by laser diffraction particle size analyzer.

Device: Malvern Mastersizer Laser Analyzer 3000 with Hydro SV dispersion unit.

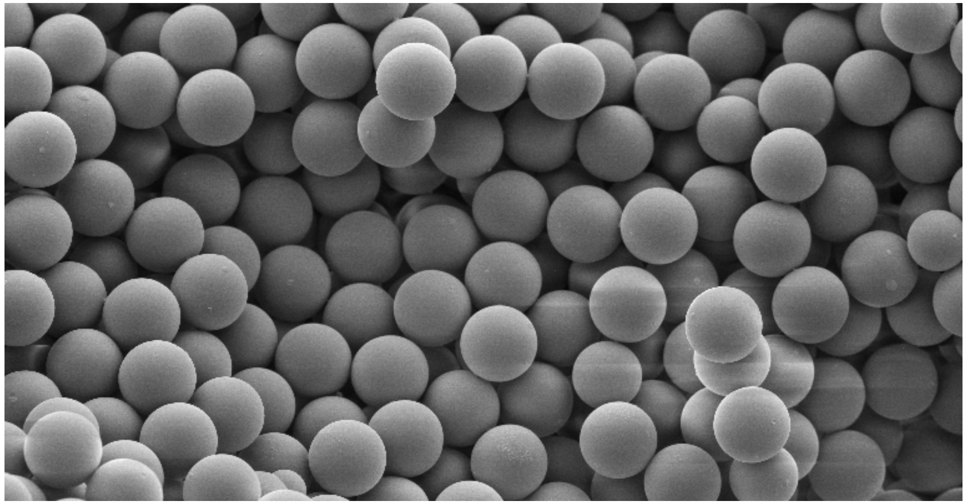

The SEM picture of 5.04µm particles packed into capillary prior to etching.
